# Humoral Response against Small Heat Shock Proteins in Parkinson’s Disease

**DOI:** 10.1371/journal.pone.0115480

**Published:** 2015-01-28

**Authors:** Ewa Papuć, Ewa Kurys-Denis, Witold Krupski, Konrad Rejdak

**Affiliations:** 1 Chair and Department of Neurology of Medical University, Lublin, Poland; 2 2nd Department of Radiology, Medical University, Lublin, Poland; Weizmann Institute of Science, ISRAEL

## Abstract

**Introduction:**

In the light of evidence for the increased heat shock proteins (HSP) expression in neurodegenerative disorders, the presence of the adaptive humoral response of the immune system can be expected. The aim of the study was to check whether Parkinson’s disease (PD) has the ability to elicit immune response against small heat shock proteins.

**Methods:**

IgG and IgM autoantibodies against alpha B-crystallin were assessed in 26 PD patients 26 healthy subjects. For the assessment of anti-HSP IgG autoantibodies serum samples from 31 parkinsonian patients and 31 healthy control subjects were collected. Serum samples from PD patients and healthy control subjects were collected twice, at baseline and after mean of 13 months follow up.

**Results:**

Both IgM and IgG autoantibodies against alpha ß-crystallin in PD patients were significantly higher compared to healthy controls (p<0.05). We also found statistically significant increase in antibodies titers against alpha ß-crystallin over the time of 13 months, both for IgG (p = 0.021) and for IgM (p<0.0001). Additionally, PD patients presented higher levels of anti-HSP IgG autoantibodies than healthy controls (p = 0.02).

**Conclusions:**

Increase of IgG and IgM autoantibodies against alpha B-crystallin in PD patients over time may suggest their involvement in the disease pathogenesis and progression. Further studies are required to confirm the role of this antibody as a biomarker of the disease progression.

## Introduction

Heat shock proteins are functionally and immunologically highly conserved molecules present in almost all living organisms [1]. Their expression in the cell increases under the circumstances that are potentially harmful to cells, for example, high temperature. This increased HSP expression is present in cells exposed to mild stress and this protects them against subsequent stress. However, in cells subjected to severe stress, HSP promote apoptosis.

HSP do not only protect proteins from denaturation, they also have immune functions. HSPs control the correct folding of nascent and denaturated proteins, are responsible for promoting the degradation of denatured proteins, they also help to maintain cellular homeostasis and protect from cell death through a mechanism called thermotolerance [2].

Bacterial and human Hsp share considerable homology and antibodies or T-cells that recognize microbial Hsp as immunodominant antigens often cross-react with human Hsp [3]. Based on molecular-weight, HSPs can be divided into the large (HSP100: 100–110 kDa and HSP 90: 75–96 kDa), intermediate (HSP 70: 66–78 kDa, HSP60, and HSP40), and small (sHSP: 8.5–40 kDa) subfamilies. Members of HSP family display dual activity depending on their intra- or extracellular distribution. Intracellular HSPs mainly play a protective role. Extracellular membrane-bound HSPs mediate immunological functions.

### Anti-HSP-60 autoantibodies

Anti-60 kD heat shock protein (Hsp60) antibodies are present in serum of healthy human subjects [4,5,6], also in samples of patients with atherosclerosis [7] and other vascular disorders [6,8]. Anti-HSP autoantibodies may be also found in patients with inflammatory and autoimmune disorders [2,9]. As atherosclerosis is considered to be connected with inflammation it may by hypothesized that different other inflammatory disorders, including PD, may be accompanied by the presence of anti-HSP antibodies. Thus we wanted to check whether this chronic neurodegenerative process has the ability to elicit immune response against small heat shock proteins, subsequent to their increased expression. This is especially interesting in the light of evidence for underlying chronic inflammatory process present in PD [10].

### Alpha B-crystallin

There is some evidence that different heat shock proteins may suppress alpha-synuclei (αSyn) formation. Its aggregation may be suppressed by the molecular chaperone Hsp70 [11] or by alpha B-crystallin [12]. Alpha B-crystallin is a small heat-shock protein (sHsp) that is colocalized with aSyn in Lewy bodies, which are pathological hallmarks of Parkinson’s disease. There is evidence that alpha B-crystallin is an inhibitor of alpha-synuclein amyloid fibril formation in vitro [13]. Alpha B-crystallin probably plays a protective role in preventing the toxicity associated with improper protein misfolding, although the interaction of alpha B-crystallin with amyloid beta (Aβ) has produced confounding hypotheses. Stege et al. [14] observed that alpha B-crystallin inhibited the formation of mature Aβ fibrils and so concluded that alpha B-crystallin stabilizes Aβ into a more toxic β-sheet-rich oligomeric form. In contrast, Raman et al. [15], Wilhelmus et al. [16] and Dehle [17] reported that alpha B-crystallin inhibited fibril formation by Aβ, which has a neuroprotective effect. Thus, it is still unclear whether such inhibition is neuroprotective [14,16]. Alpha B-crystallin is upregulated in response to a range of stress stimuli and clinical disorders including Alzheimer’s disease, transmissible spongiform encephalopathies, dementia with Lewy bodies, and Parkinson’s disease [18].

In this study we check whether increased expression of alpha B-crystallin induces humoral immune response against this small heat shock protein.

## Materials and Methods

For assessment of alpha B-crystallin, IgG and IgM autoantibodies 26 PD patients in advanced clinical stage (Hoehn-Yahr scale 3–4) consecutively admitted to the Department of Neurology of Medical University of Lublin, Poland were enrolled. Serum samples from PD patients were collected twice, at baseline (time-point #1) and after mean of 13 months follow up (time-point #2). In addition, serum samples from 26 healthy controls matched for age and gender were assessed for the presence of IgG and IgM autoantibodies against alpha ß-crystallin, samples were collected twice, at baseline (time-point #1) and after mean of 13 months follow up (time-point #2). IgG and IgM autoantibodies against alpha B-crystallin were measured by a commercially available ELISA system according to the instructions of the manufacturer (Mediagnost, Germany). All analysis were performed in duplicate. The ELISA (E100) uses an internal standard pool serum for calculation of antibody titers and employing microplates coated with myelin-specific proteins purified from bovine brain. The autoantibody titer was calculated after the subtraction of nonspecific binding and blanks. The titers were estimated on the base of calibration curve of autoantibody standards and expressed in Mediagnost Units per milliliter (MU/mL).

For the assessment of anti-HSP IgG autoantibodies serum samples from 31 parkinsonian patients and 31 healthy control subjects were collected twice, at baseline (time-point #1) and also after mean of 13 months follow up (time-point #2).

We used enzyme-linked immunosorbent assay for measuring anti-HSP antibodies, which is a validated method (ADI-EKS-650, Enzo Life Sciences). Concentration values were expressed in ng/ml. Assay Designs Anti-Human Hsp60 (total) ELISA Kit uses recombinant human Hsp60 bound to the wells of the immunoassay plate to bind anti-human Hsp60 antibodies present in human serum.

Blood were collected between 8:00 and 10:00 a.m., transferred to the lab on ice, centrifuged and serum was stored at −70◦C within 60 minutes thereafter. The study was approved by the local Ethics Committee of Medical University of Lublin, Poland, and all study participants gave written informed consent for study participation.

## Statistical Analysis

Antibodies titer difference between both time-points and different investigated subgroups were estimated with the use of Anova single factor test. P value < 0,05 was considered statistically significant (two sided). Statistical calculations were done with the usage of InStat GraphPad Software Inc, CA.

## Results

### Antibodies against alpha B-crystallin

We confirmed the presence of IgM and IgG autoantibodies against alpha B-crystallin in investigated group of PD patients and observed statistically significant higher levels of both IgG and IgM autoantibodies titers in parkinsonian patients compared to healthy control subjects (both at time point 1 and at time point 2) (p<0.05).

We also found statistically significant increase in antibodies titers against alpha-B-crystallin in parkinsonian patients over the investigated period of time, both for IgG (p<0.05) and for IgM (p<0.05). No statistically significant differences were found in antibodies titers against alpha B crystallin in healthy control group over the investigated period of 13 months, neither for IgG nor for IgM. Demographical, clinical and biochemical characteristics of the study population are shown in the Table 1, Table 2 and Table 3.

**Table 1:** Demographic data of the study population.

Demographic characteristic of study group	PD patients	Control group I (for anti- alpha B crystalline antibodies)	Control group II (for anti—HSP 60 antibodies)
Subjects (female/male)	n = 31 (15/16)	n = 26 (12/14)	n = 31 (15/16)
Age [years] ±SD	61.2±5.7	58.2±6.1	58.04±5.6
Age of PD onset [years]	53.38±4.15	NA	NA
Disease duration [years] (range)	7.76±3.14 (4–12.5)	NA	NA
MMSE [0–30]	23.6±0.9	27.5±1.1	28.65±1.2
Time period between 1st and 2^nd^ sample collection in months Mean±SD (range)	13.1±2.8 (10–17)	11.8±1.8 (10–15)	12.35±1.9 (10–16)

MMSE—Mini Mental State Examination. NA not applicable. Data are presented as means with standard deviation (SD).

**Table 2:** Clinical data of the study population.

Clinical data	PD patients
Mean off UPDRS	39.3±6.3
Mean on UPDRS	30.7±5.2
Mean Hoehn-Yahr score	3.7±0.6
Mean levodopa equivalent (mg)	1913.1
Predominant tremor (n)	17
Predominant axial features (n)	14

UPDRS-Unified Parkinson’s Disease Rating Scale.

**Table 3:** Antibodies titers in investigated subgroups.

Biochemical data	PD Time point #1	PD Time point #2	Control Time point #1	Control Time point #2	ANOVA, *p* value
**Anti-alpha B-crystallin** IgG titer [MU/mL], mean ±SD	1.64±0.37*	2.96±0.98 * †	1.04±0.68	1.06±0.73	p<0.05
**Anti-Alpha B-crystallin** IgM titer [MU/mL], mean ±SD	8.41±1.39 *	9.17±1.76* †	5.67±2.15	5,76±1.53	p<0.05
**Anti HSP-60** IgG titer [ng/mL], mean ±SD	2.16±1.05*	2.52±0.88*	1.64±0.8	1.61±0.76	p<0.05

Data are presented as means with standard deviation (SD).

* p<0.05, significant difference in comparison to control;

† p<0.05, significant difference between Time-point #2 in comparison to Time-point #1, Tukey-Kramer post-hoc test.

### Anti-HSP autoantibodies

PD patients presented statistically significant higher levels of anti-HSP IgG autoantibodies than healthy controls, both at time point 1 (p<0.05) and time point 2 (p<0.05). Neither in a group of PD patients nor in healthy control patients, statistically significant differences were found in the anti-HSP60 IgG autoantibodies over the investigated period of time (between time point 1 and time point 2) (p>0.1).

## Discussion

### Autoantibodies against alpha B-crystallin

Increase of IgG and IgM antibodies against alpha ß-crystallin with longer disease duration, may suggest the increasing role of this protein in prevention of alpha-synuclein amyloid fibril formation with longer lasting neurodegenerative process. It is known that alpha-synuclein-positive inclusions are not only found in neurons but also in oligodendrocytes and astrocytes of Lewy body disease subjects [19,20]. In these cells, number of alpha-synuclein-positive inclusions have been shown to correlate positively with the extent of neurodegeneration in the substantia nigra [19]. It is possible that with more widespread neurodegenerative process and the presence of numerous alpha-synuclein deposits, the expression of protein alpha B-crystallin increases. Progressive neuronal death and release of these proteins probably induces immune response, which is reflected by the increase in the antibodies titers against alpha B-crystallin. This would support the hypothesis that alpha B-crystallin may play a protective role against neurotoxicity.

It is however worth noting that the assessment of humoral response against alpha B crystallin in different inflammatory disorders should be interpreted cautiously, especially in the light of data presented by Rothbard et al [21]. The authors concluded in their study, performed on multiple sclerosis (MS) patients, that small HSPs (including alpha B—crystallin) bind immunoglobulins (Igs) with high affinity, and in fact are receptors of the Igs, not the antigens for them. The authors [21] proved that antibodies against alpha B crystallin cross-react with different other small HSPs which contradict the hypothesis that alpha B crystallin should be treated as an autoantigen. That is why, the assessment of humoral response in different inflammatory disorders may be difficult, as majority of immnoassays are based on typical antibody-antigen interaction, and they do not consider the possibility of the antigen binding the antibody [21]. Rothbard’s study suggest that alpha B crystallin may be a ligand which binds different antibodies regardless of their specifity, but does not elicit specific immune response. Whether these results presented by Rothbard et al. are disease specific (MS), or can be generalized to other disorders involving inflammation, should be a subject of further research. It is worth noting that Rothbard et al. observed equivalent Igs reactivity for both sera of MS patients and healthy controls, contrary to our study, where we found differences in both IgG and IgM reactivity between parkinsonian patients and healthy controls.

### Anti-HSP autoantibodies

We hypothesize that possible reason for the increased presence of anti-HSP60 autoantibodies in serum of parkinsonian patients is neurodegenerative process. The increased expression of different HSP due to neurodegenerative stress, results in significantly higher auto-antibodies titers in parkinsonian patients. The exact mechanism explaining the role of HSPs in autoimmunity has been explained by Raijaih [22]. HSPs are highly conserved among microbial agents and among mammals. Mammalian HSPs may be targets of immune response, as immunoregulatory cells primed against microbial HSPs, may be autoreactive and recognize epitopes on the human HSPs [22]. This molecular mimicry phenomenon is probably augmented by increased expression of HSPs under stress, we assume that also by chronic neurodegenerative process. Under stress, previously hidden antigenic determinants become unmasked, and that can initiate and led to augmentation of autoimmune reactivity.

Obviously, the influence on autoimmunity, of factors other than chronic neurodegenerative process cannot be excluded. There is multiple evidence that HSPs are involved in the regulation of different autoimmune disorders [22,23,24,25]. Autoimmune disorders have all genetic background, nevertheless in individuals, who are genetically primed, additional factors may trigger the onset of the disease. There is emerging evidence that these factors are particularly the viruses, which realize their effects by influencing activity of HSPs [25]. That is why we compared our PD patients with healthy control people, finding significantly higher levels of anti-HSP60 autoantibodies in people with neurodegenerative disorder. Considering the fact that these two groups have probably comparable exposition to viral infections, we still hypothesize that chronic neurodegenerative process has its own impact on eliciting immune response.

The presence of anti-HSP autoantibodies is healthy people and in different disorders remains unclear. They could be cross-reacting antibodies induced by bacterial infections [9] or real autoantibodies [3]. It is often hypothesized that the carriage of high anti-Hsp60 autoantibodies may be a part of natural antibody repertoire, which can be an inherited trait and the cumulative antibody inducing effects of multiple infections add to this trait [4]. Natural antibodies refer to antibodies that are present in the serum of healthy individuals without overt immunization or infection [26]. Thus the presence of anti-self Hsp autoantibodies may be integral part of the normal immune function, playing role in self-protection and regulation of autoimmunity. Basic features of natural autoantibodies include polyreactivity, life-long stability, connectivity, presence in samples of newborn organisms without antigenic stimulation (indicating germ-line encoding) and low affinity [27]. In humans natural autoantibodies may belong to IgG, IgM and IgA isotypes with the predominance of IgG [27]. In this study we confirmed the stability of anti-HSP 60 autoantobodies in PD and healthy control subjects over a period of 1 year. Our data are in accordance with data regarding other disorders. The stability of anti-Hsp60 autoantibody titers in coronary heart disease over long observation period [6] also supports the hypothesis that anti-Hsp60 autoantibodies might belong to the group of natural autoantibodies. Varbiro et al. also document the stability of IgG antibody reactivity directed toward the conserved Hsp60 through an observation period of five years in healthy humans [4]. Also Lacroix-Desmases et al. [27] observed stability of the natural self-reactive IgM and IgG antibodies with aging.

**Fig 1:**
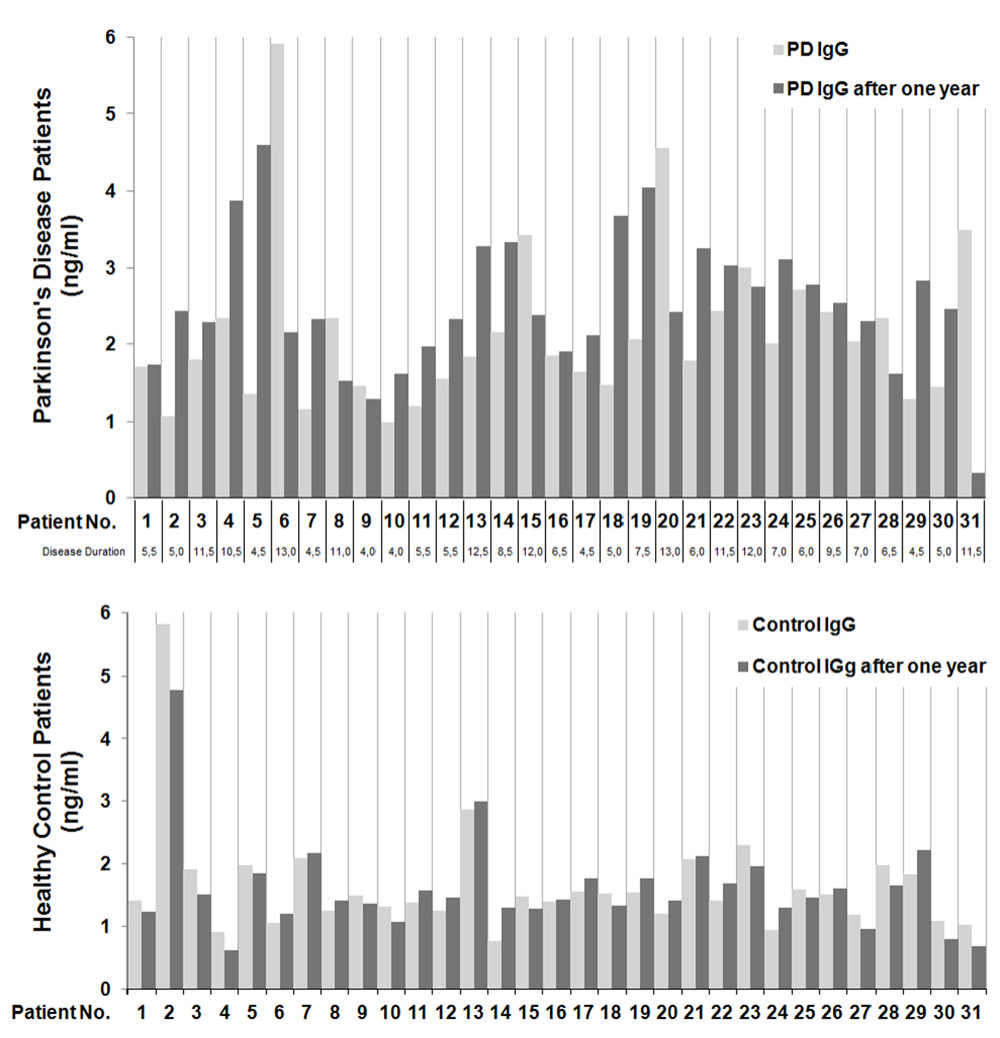
Variability of anti-HSP60 antibodies titers in investigated groups of patients.

Although autoantibody levels against different conserved proteins remained stable with age, large inter-individual differences were observed with regard to relativities [28]. Mouthon proved that in newborns and adults autoreactive repertoires are highly conserved among individuals [29]. Varbiro et al. revealed that IgG reactivity toward Hsp60 may also be characteristic for a given individual as well. Also in our sample of patients we also observed large variability of anti-HSP60 IgG antibodies titers, which is presented in Fig. 1.

The antibody IgG repertoire in serum of healthy adults was presented by Mouthon et al [29]. The authors proved that in sera of healthy people, there exist natural IgG autoantibody repertoire that are specifically selected for reactivity with a limited set of self-antigens. The unique reactivity pattern of each individual is controlled additionally by different factors, not related to IgG, this phenomenon is described as an “antibody immuno-fingerprinting” [29].

As anti-HSP 60 autoantibodies were detected both in serum of parkinsonian patients and healthy controls, we admit that these antibodies may belong to the natural autoantibodies repertoire, but chronic neurodegenerative process has additional inducing effect.

Comparison of restricted and conserved autoreactive IgG repertoire of healthy controls and PD patients may allow in the future to delineate immune response typical for PD. This in turn could be potentially helpful to find the surrogate markers for this chronic neurodegenerative disorder.

## Conclusions

Anti-HSP IgG autoantibodies belong probably to the natural auto-antibodies, as they are present in healthy people, nevertheless chronic neurodegenerative process may have additional inducing effect on humoral response involving anti-HSP autoantibodies, which is reflected by significantly higher levels of anti-HSP 60 autoantibodies in PD patients compared to healthy controls.

Increased titers of IgM and IgG autoantibodies against alpha B-crystallin in PD patients may reflect activation of humoral immune response in the course of this chronic disease, probably secondary to increased expression of this heat shock protein. Nevertheless, there is also emerging evidence that alpha B crystalin and other small HSP, is not the antigen inducing Ab response, but the receptor for the Igs [21], which can make the objective assessment of humoral response against small HSP impossible.

Increase in IgG and IgM antibodies titers against alpha B-crystallin over the investigated period of time reflects activation of the immune response, probably secondary to widespreading neurodegenerative process and may suggest the involvement of the immune system in the disease progression. Further studies are needed to assess whether these antibodies may serve as biomarkers of disease progression.
